# Diet’s rapid effects on the thyroid gland challenge unidirectional assumptions about hypothyroidism and energy balance

**DOI:** 10.1172/JCI205029

**Published:** 2026-04-15

**Authors:** Arturo Hernandez, Francesco S. Celi

**Affiliations:** 1 Center for Molecular Medicine, MaineHealth Institute for Research, MaineHealth, Scarborough, Maine, USA.; 2 Graduate School of Biomedical Sciences and Engineering, University of Maine, Orono, Maine, USA.; 3 Department of Medicine, Tufts University School of Medicine, Boston, Massachusetts, USA.; 4 Department of Medicine, UConn Health, Farmington, Connecticut, USA.

## Abstract

Thyroid hormones provide crucial regulation of energy expenditure through their effects on thermogenesis, lipid metabolism, and mitochondrial function. Given their close relationship with overall metabolism, a deficit in thyroid hormones fittingly leads to a positive energy balance and weight gain. The direction of this process may also operate in reverse, according to recent research from Rampy et al. in this issue of JCI. These investigators found that mice introduced to a high-fat, high-sugar diet developed marked short-term intracellular stress and functional impairment in the thyroid gland, leading to alterations in serum thyroid hormone levels prior to measurable weight gain. This finding opens the possibility that thyroid dysfunction originates from persistent damage to the thyroid gland caused by sustained overnutrition.

## Introduction

The impact of thyroid hormone states on metabolism and energy expenditure is well known. The active thyroid hormone, T3 (3,5,3′-triiodothyronine) promotes mitochondrial respiration and overall metabolic rate (1) and regulates hepatic lipid metabolism (2). T3 stimulates thermogenesis in brown adipose tissue (3), futile cycles (1), and lipogenesis and thermogenic capacity (known as “beiging”) in white adipose tissue (4). Furthermore, T3 also increases energy expenditure through central actions on hypothalamic circuits (5, 6). Because of these roles, changes in weight and temperature sensitivity are primary clinical manifestations of human thyroid dysfunction. While hyperthyroidism classically causes weight loss, muscle wasting, and heat intolerance, hypothyroidism leads to a decrease in energy expenditure, increased adiposity, and cold intolerance and facilitates weight gain (7).

## Rapid dietary effects on thyroid gland function

**Figure 1:**
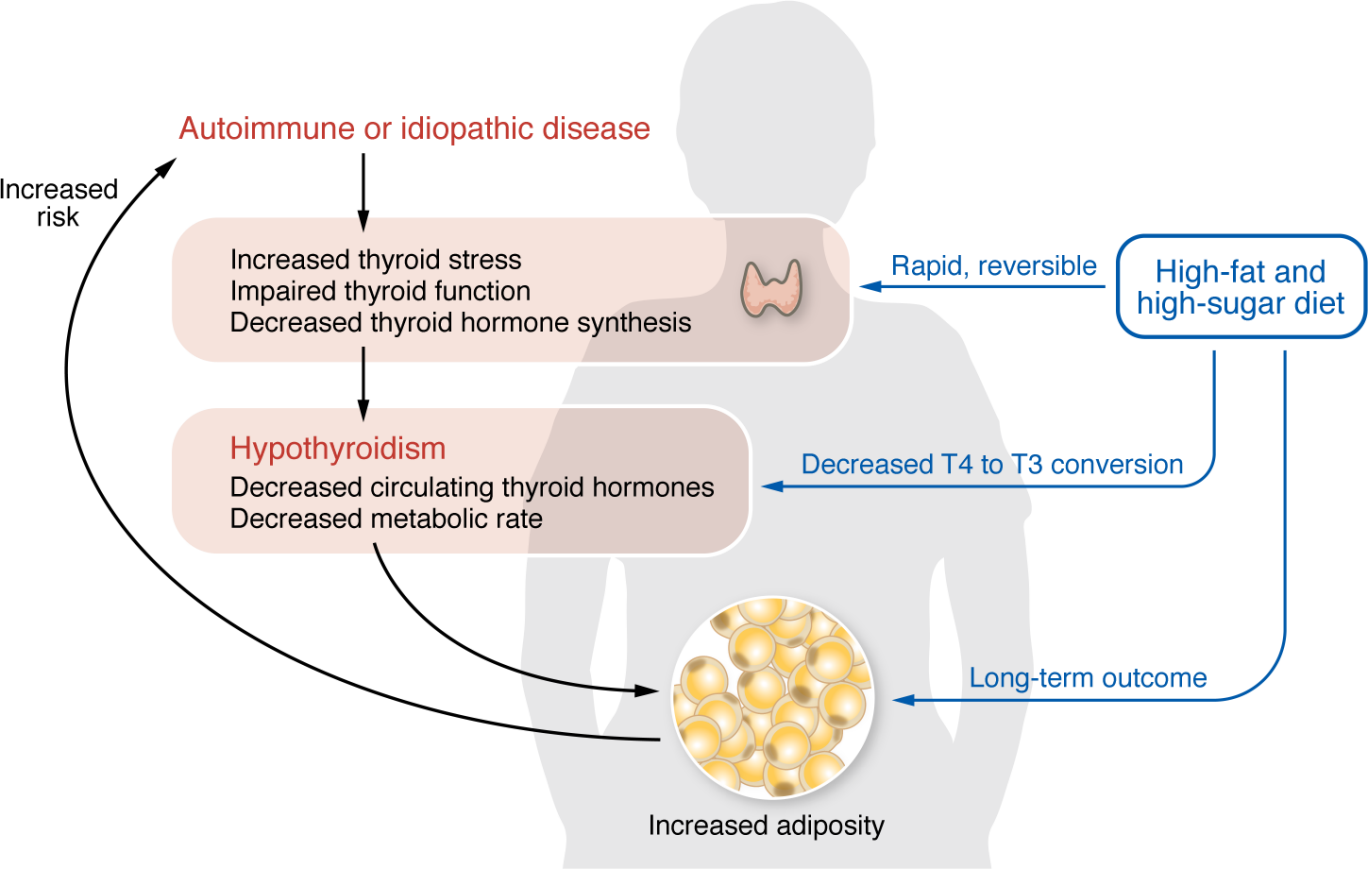
A high-fat, high-sugar diet rapidly causes cellular stress and impaired function of the thyroid gland. Although obesity is a risk factor for autoimmune thyroid disease, the prevalent paradigm is that impaired thyroid function leads to hypothyroidism and increased adiposity and weight gain, a potential symptom of a malfunctioning thyroid gland (left side, highlighted in red). The work of Rampy et al. (8) suggests that an unhealthy diet rapidly impairs the thyroid gland, as well as T4-to-T3 conversion in peripheral tissues, before hypothyroidism and weight gain are evident (right side, highlighted in blue). This work provides a complementary (or even competing) paradigm for the etiology of thyroid dysfunction, a highly prevalent condition.

The functions of thyroid hormones described above underlie the general assumption that there is a causal, unidirectional relationship linking thyroid hormones with energy balance and associated body composition. Under this assumption, a hypofunctional thyroid gland (whether due to autoimmune thyroid disease like Hashimoto’s, other conditions, or surgical removal of the gland) will cause hypothyroidism, and the decrease in circulating thyroid hormones will lead to a lower metabolic rate and a tendency to gain weight over time (Figure 1, highlighted in red). However, work published in the present issue of the Journal of Clinical Investigation (8) partly challenges this unidirectional assumption about the etiology of hypothyroidism. A study from the group of Nancy Carrasco suggests that the causal relationship between thyroid hormones and energy balance may also work in the opposite direction (8) (Figure 1, highlighted in blue). To model conditions of “overnutrition,” mice were fed a hypercaloric high fat diet and provided with 5% sucrose in the drinking water. Rampy et al. observed that mice on the hypercaloric diet exhibited changes in circulating thyroid hormones that are similar to the ones observed in the early phase of primary hypothyroidism in humans, including reduced serum thyroxine (T4) and elevated thyrotropin (TSH), while T3 levels remained unchanged. Rampy et al. associated these abnormal parameters with major cellular abnormalities in the thyroid gland, including goiter, reduced T4 content, and increased granularity and vascularity. After 6 weeks on the hypercaloric diet, thyroid glands from these mice exhibited broad and profound changes in gene expression, with more than 6,000 differentially expressed genes associated primarily with cell proliferation and TSH responsiveness, as well as with mitochondrial and endoplasmic reticulum stress. Furthermore, in preliminary examinations of human thyroid samples, the authors observed a significant correlation between thyroid gland vascularization, a TSH-dependent endpoint, and body mass index, consistent with the possibility that the effects observed on mice also occur in humans.

Although serum hypothyroidism has been noted in genetic and diet-induced models of obesity, analysis of the thyroid gland has been largely lacking. The moderate hypothyroidism observed in models of obesity has been mostly interpreted as a result of leptin resistance and partial central hypothyroidism (9), as leptin is able to stimulate the hypothalamic expression of thyrotropin releasing hormone (10, 11), the central driver of thyroid axis physiology. In the present work (8), Rampy et al. showed that an obesogenic diet directly and profoundly affects the thyroid gland and thyroid hormone biosynthesis. The authors also observed a decrease in the activity of brown fat D2 deiodinase, an enzyme that converts T4 to T3 in multiple peripheral tissues of relevance to thyroid axis physiology and metabolic function including the hypothalamus, the pituitary, adipose tissue, and, in humans, skeletal muscle (12, 13). Given the broad role of thyroid hormones as determinants of metabolism, Rampy et al.’s work has immediate, broad implications for metabolic studies. It brings unique and critical attention to likely changes in thyroid gland function and peripheral thyroid hormone metabolism that affect interpretation of data from animal research models utilizing high-fat diets. It is likely that a severely impaired thyroid gland is a critical contributor to the metabolic phenotypes observed in diet-induced obesity studies, in which the diet is often provided to experimental rodents for longer periods than the 6 weeks used in the present work.

The most remarkable observation in the work of Rampy et al. (8) is that several of the thyroid gland alterations were noticeable after just 3 weeks on the diet, and T4 content was significantly reduced after only 10 days on the diet. These changes occurred before any changes in weight or circulating leptin were measurable. Limitations of this study include its lack of sufficient measurements of indices of thyroid hormone activation and action (14) and receptor occupancy (15), which would have provided a helpful assessment of thyroid hormone status in multiple metabolic tissues, as well as the absence of dynamic measurement of thyroid activity by scintigraphy, which would allow further assessment of the degree of thyroid dysfunction. Despite these limitations, the main observation of this work prompts a highly relevant and intriguing clinical question about the etiology of hypothyroidism: Is inadequate diet the main or even the sole initial cause of thyroid dysfunction? Rampy et al.’s findings support this possibility not only in cases of idiopathic hypothyroidism but also in patients with Hashimoto’s thyroiditis, a major cause of hypothyroidism, for which risk is substantially elevated in obese individuals (16). It is conceivable that, over time, the diet-induced stress observed in follicular cells may prompt a state of inflammation in the thyroid, triggering the autoimmune response, which is the hallmark of Hashimoto’s thyroiditis. Thus, some of these patients may have already functionally impaired thyroid glands due to unhealthy diets and low-grade inflammation, even before clinical manifestations of hypothyroidism are evident or elevated antithyroid antibodies are present.

Rampy et al.’s observation of the obesogenic diet’s relatively rapid action on the thyroid gland suggests the existence of sensitive mechanisms by which the organism perceives the aberrant diet, impacting the thyroid. Although these mechanisms remain to be elucidated, some of them may potentially involve the way thyrocytes sense the abnormal availability and subsequent utilization of fuel substrates for cell metabolism. They may also include inflammatory signals or adipokines that start to change after a few days on the diet, or metabolic sensing by the hypothalamus influencing the central regulation of TSH output. Although Rampy et al. did not investigate glucose-insulin homeostasis in their experiment, it is likely that mice on this obesogenic diet start developing insulin resistance, which may directly or indirectly affect the thyroid. This possibility may partly explain the established clinical correlations between type 2 diabetes and hypothyroidism (17) and impaired T4 to T3 conversion in peripheral tissues (18). In this regard, it will be of substantial clinical value for future studies to delineate whether the harmful effects on the thyroid gland can be recapitulated by the high fat diet or the sugary water independently, or whether the combination of both interventions is required for the deleterious effects.

## Clinical implications

On the bright side, Rampy et al. showed that the undesirable consequences of the obesogenic diet for the thyroid were largely reversible (8), an observation consistent with the finding that weight loss in humans may affect peripheral T4 to T3 conversion (19). After switching back to a normal diet for 6 weeks, mice in this study shifted back toward a normalized body weight, thyroid gland vascularity, and thyroidal T4 and thyroglobulin content. Significant goiter and elevated TSH still persisted at the 6-week time point, and it will be of critical interest to determine whether these parameters would have also normalized after a longer period of time on the normal diet, or whether they would persist as a permanent physiological signature of past high-fat diet exposure and future susceptibility to thyroid disease. Yet, the reversibility of most of the pathological effects of such diet on the thyroid gland is encouraging. It would indicate that dietary interventions may aid in the treatment of some hypothyroid patients, provided that this phenomenon fully applies to humans as suggested by the clinical data presented by the authors and by published studies that have revealed a positive correlation between body mass index and TSH levels (20).

At the very least, the work of Rampy et al. convincingly supports the existence of a causal, bidirectional relationship between hypothyroidism and positive energy balance. According to this new paradigm, not only would a reduction of circulating levels of thyroid hormones reduce metabolic rate and cause increased adiposity and weight gain, but an obesogenic diet would cause thyroid gland stress and an impairment of thyroid hormone biosynthesis. These thyroid gland abnormalities may appear rather rapidly, before any significant weight gain is evident, and could be potentially noticeable as an individualized sub-clinical reduction in circulating T4. Appropriately designed studies in humans are needed to define to what extent the findings in this work (8) are translatable to the clinical setting. For the time being, Rampy et al.’s insightful study opens the possibility that a substantial proportion of cases of hypothyroidism primarily originates from sustained dietary fat and sugar excess, which would initiate a self-reinforcing pathophysiological loop of a stressed, hypofunctional thyroid gland and a positive energy balance. In the context of current human diets, the steadily increasing prevalence of hypothyroidism in recent decades, and the tight relationship between metabolic rate and thyroid hormones, it will not be particularly surprising if we eventually discover that hypothyroidism does start with your diet.

## Conflict of interest

The authors have declared that no conflict of interest exits.

## Funding support

This work is the result of NIH funding, in whole or in part, and is subject to the NIH Public Access Policy. Through acceptance of this federal funding, the NIH has been given a right to make the work publicly available in PubMed Central.

- National Institute of Diabetes, Digestive and Kidney Diseases (DK095908) (to AH).
- National Institute of Diabetes, Digestive and Kidney Diseases (DK140455) (to FSC).

## Version 1 04/15/2026

Electronic publication
